# Drug-associated tongue discoloration: a comprehensive assessment of USFDA adverse event reporting system using disproportionality analysis

**DOI:** 10.1038/s41405-026-00402-7

**Published:** 2026-02-02

**Authors:** Kannan Sridharan, Gowri Sivaramakrishnan

**Affiliations:** 1 Department of Pharmacology & Therapeutics, College of Medicine & Health Sciences, Arabian Gulf University, Manama, Kingdom of Bahrain; 2 Bahrain Defence Force Royal Medical Services, Riffa, Kingdom of Bahrain

**Keywords:** Xerostomia, Aphthous stomatitis

## Abstract

**Background:**

Drug-induced tongue discoloration can impact quality of life but remain under-recognized. While several antimicrobials are implicated, comprehensive signals across many drug classes remain poorly characterized.

**Methods:**

Reports of tongue discoloration, strawberry tongue, and black hairy tongue were extracted from the USFDA Adverse Event Reporting System from 2004 to 2024. Disproportionality analysis was conducted using reporting odds ratio, proportional reporting ratio, Bayesian confidence propagation neural network, and multi-item gamma Poisson shrinker to detect associations between implicated drugs and tongue conditions.

**Results:**

A total of 2352 reports were analyzed. Drugs consistently associated with tongue discoloration by both frequentist and Bayesian analyses included clarithromycin, metronidazole, linezolid, amoxicillin, and fluticasone. Flucloxacillin and immunoglobulin G emerged as risks for strawberry tongue. Meclizine showed a signal for black hairy tongue across methods, whereas other drugs appeared risk factors using only frequentist tests. Demographic patterns varied by condition.

**Conclusion:**

This comprehensive signal detection revealed several high-priority drug-tongue condition associations using large-scale pharmacovigilance data. Findings carry implications for guiding clinical practice through enhancing adverse effect monitoring for high-risk medications. Priority signals merit validation through further research to optimize benefit-risk assessment and reduce preventable tongue disorders.

## Introduction

Drug-associated tongue discoloration is a significant oral hard tissue alteration that can impact taste sensation and quality of life. Tongue discoloration can be broadly classified into two categories based on the mechanism of color change: intrinsic and extrinsic discoloration. Intrinsic discoloration occurs when chromogenic substances are incorporated into the tongue tissue structure during development or through systemic absorption, resulting in color changes that originate from within the tissue itself [1]. This type involves actual alteration of the tongue’s cellular components and extracellular matrix. In contrast, extrinsic discoloration results from the external deposition of chromogenic materials on the tongue surface, which can often be removed through mechanical cleaning or appropriate oral hygiene measures. There are several types of tongue discoloration, including strawberry, black, green, and yellow variants. Black hairy tongue, also called lingua villosa nigra, results from a failure of desquamation and papillae hypertrophy in the tongue, altering its color [2]. Similarly, prominence and fungiform papillae hypertrophy cause strawberry tongue. The prevalence of black hairy tongue varies widely, ranging from 0.6% to 11.3% [3, 4]. Identified risk factors include male sex, older age, smoking, HIV positivity, and cancer [4].

The pathophysiology is not fully established, but reports indicate that drug or metabolite deposition leads to discoloration [5]. Reaction of deposited drugs with iron and melanin alters oral mucosa color [6]. Patients with black hairy tongue exhibit severe oral dysbiosis characterized by a Proteobacteria-dominated microbiome [7].

The United States Food and Drug Administration Adverse Event Reporting System (FAERS) collects adverse event reports from healthcare personnel via spontaneous reporting [8]. Disproportionality analysis applies statistical tests to pharmacovigilance databases to identify potential adverse event signals for drugs or devices [9]. To date, one Dutch study assessed prescribed drug-associated tongue disorders, implicating seven Anatomical Therapeutic Chemical (ATC) drug classes, with over 52% attributed to systemic antimicrobials and 19% to dermatological drugs [10]. Reported antimicrobial causes include minocycline, doxycycline, erythromycin, linezolid, amoxicillin-clavulanate, metronidazole, and piperacillin-tazobactam [10]. However, data on drugs linked to tongue discoloration are limited, motivating this study. This study was carried out to: (1) identify drugs associated with tongue discoloration using the FAERS database; (2) perform disproportionality analysis to detect safety signals; (3) characterize patient demographics; and (4) provide evidence-based information for clinical practice regarding drug-associated tongue discoloration risks.

## Methods

### Data source

We extracted data from the FAERS using the Standardized Medical Dictionary for Regulatory Activities (MedDRA), listed lowest level term, pre-determined and pre-validated set of terms coded by expert panel in MedDRA [11]. The data obtained was collected pertaining to the spontaneously reported adverse events for the period of March 2004 until the second quarter of 2024 (82 quarters).

### Data processing

The FAERS database was queried separately using the lowest level terms “tongue discoloration”, “strawberry tongue”, and “tongue black hairy” [12]. We adhered to the USFDA recommendations for deduplicating the adverse event reports. The reports were first sorted out using the Case _ ID for identifying duplicate records and those with the highest FDA _ DT (or Individual Safety Reports number) were retained while excluding other reports. While submitting the spontaneous reports, the USFDA allows the healthcare personnel to choose one of the roles of suspicion between the drug-adverse event pair into one of the following categories: primary suspect, secondary suspect, interacting, or concomitant. Only the reports where the association between the suspected drug and the adverse event was considered primary were included in this study. For drugs associated with positive signals, their demographic characteristics (age, gender, year and country of reporting) were collected.

### Data mining algorithms

We employed disproportionality analysis using “case-non-case studies” approach for identifying signals between drugs and tongue discoloration. This approach is based on the comparison between the exposure to drug of interest to a specific adverse drug event (cases) with those of other reported events (non-cases) [13]. We used the Openvigil 2.1 platform for obtaining the appropriate data that included demographic details and drug-related information. We employed four data mining algorithms for signal detection that are classified into frequentist and Bayesian approaches. The frequentist measures used in this study were reporting odds ratio (ROR) and proportional reporting ratio (PRR) [14]. A Signal was detected as the disproportion in the distribution of number of cases was all three criteria (Evans’) are met: PRR ≥ 2, Chi-square (*χ*^2^) ≥ 4 and at least 3 cases should exist for each drug-tongue discoloration pair [14]. These signal detection criteria are standardized metrics widely accepted in pharmacovigilance practice, recommended by both the World Health Organization and regulatory authorities, including the US FDA to ensure consistent and reliable signal detection across pharmacovigilance studies. The greater the point estimate of ROR, the stronger the disproportion. Amongst the Bayesian approaches, Bayesian confidence propagation neural network (BCPNN), and multi-item gamma Poisson shrinker (MGPS) were considered in this study [14]. In BCPNN, information component (IC) was the signal detection measure as calculated by the logarithmic ratio of joint probability of drug and adverse event to the product of individual probabilities of drug and adverse drug event. IC represents the additional information obtained in eliminating the uncertain relationship between the drug of interest and adverse event, and to be considered for a signal, the lower limit of 95% CI (IC025) should exceed zero. Empirical Bayes Gamma Mixture (EBGM), calculated as the geometric mean of the empirical Bayes posterior distribution of the true report ratio. A signal was considered if the lower limit of 95% CI of EBGM05 exceeded 2. While discordance between frequentist and Bayesian signals can occur, Bayesian results are more stable and robust. Thus, even when frequentist measures were positive, signals were not considered valid unless supported by positive Bayesian measures. Only the generic names of the drugs were considered. We adhered to the first level of hierarchical coding as stated in the ATC classification by World Health Organization.

### Statistical analysis

Descriptive statistics were used for representing demographic variables. Mean (SD) was used for numerical variables and proportion (%) was used for representing categorical variables. SPSS (IBM Corp. released 2020. IBM SPSS Statistics for Windows, Version 27.0. Armonk, NY: IBM Corp.) was used for statistical analysis.

### Ethics approval

This study was carried out with the reported data available in public domain and does not require ethics approval as stipulated in the guidelines.

## Results

### Search results

**Fig. 1:**
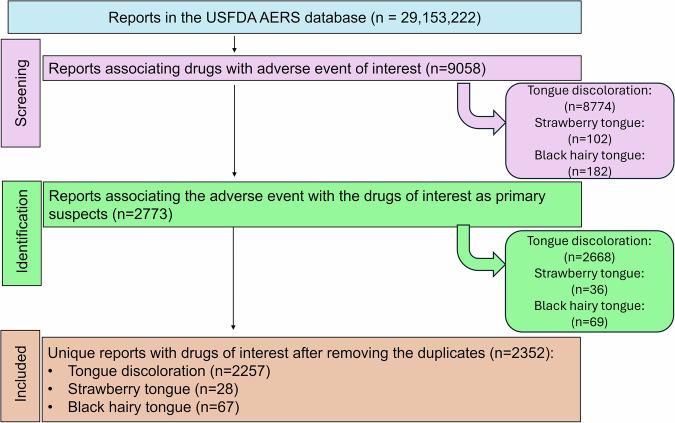
Study flow diagram. A total of 2352 unique reports were included in the final analysis.

A total of 29,153,222 reports were available in the FAERS of which 2352 unique reports were included for the analysis (Fig. 1). The demographic characteristics of the patients reported for tongue discoloration, strawberry tongue, and black hairy tongue are summarized in Table 1. Tooth discoloration and black hairy tongue were observed in middle-aged individuals, while strawberry tongue was observed mainly in adolescents and children. Female preponderance was observed for all the adverse events of interest.

**Table 1:** Demographic characteristics of patients listed in unique reports.

Characteristics		Tongue discoloration (*n* = 2257)	Strawberry tongue (*n* = 28)	Black hairy tongue (*n* = 67)
Age groups [*n* (%)]	**<18**	69 (3.1)	15 (53.6)	2 (3)
	**≥18 to** <**45**	287 (12.7)	6 (21.4)	10 (14.9)
	**≥45 to** <**65**	535 (23.7)	1 (3.6)	22 (32.8)
	**≥65**	498 (22.1)	1 (3.6)	13 (19.4)
	**Not specified**	868 (38.5)	5 (17.8)	20 (29.9)
Quantitative age (years)	**Mean ± SD**	55.4 ± 19.8	16.5 ± 19.2	56.1 ± 18.5
	**Median (range)**	58 (0-97)	12 (1-76)	56 (2-89)
Gender [*n* (%)]	**Male**	605 (26.8)	6 (21.4)	28 (41.8)
	**Female**	1461 (64.7)	20 (71.4)	34 (50.7)
	**Unknown**	191 (8.5)	2 (7.2)	5 (7.5)
Reporting top countries		USA, CA, FR	USA, CA	USA

*USA* The United States of America, *FR* France, *CA* Canada.

### Signal detection measures for drug-associated tongue discoloration

Antimicrobial agents emerged as the predominant drug class associated with tongue discoloration, with 23 individual antimicrobials demonstrating positive signals using both frequentist and Bayesian measures (Table 2). The strongest associations were observed with: Macrolide antibiotics (erythromycin, clarithromycin, azithromycin); Beta-lactam antibiotics (amoxicillin, cefuroxime, cefdinir); and Tetracycline-class antibiotics (doxycycline, tetracycline). Beyond antimicrobials, significant signals were detected for the following key drugs: Cardiovascular medications (clonidine, aliskiren, indapamide); Respiratory drugs (theophylline, budesonide, fluticasone); Gastrointestinal agents (bismuth compounds, lansoprazole); and Topical antiseptics (chlorhexidine, triclosan). We observed multiple entries for fluticasone, reflecting the exact terminology used in spontaneous reporting. While fluticasone propionate and fluticasone furoate are indeed different formulations, their separate listings primarily stem from the verbatim reporting patterns in FAERS rather than proven differences in risk profiles.

**Table 2:** Signal detection measures for drug-associated tongue discoloration

Drugs	ATC system	PRR	*χ*^2^	ROR	Lower limit 95% CI of ROR	Upper limit 95% CI of ROR	IC025	EBGM 05	Number of cases
Losartan	Agents acting on the renin-angiotensin system	2.1	19.1	2.1	1.5	2.9	0.8	1.5	35
Candesartan cilexetil		2.7	6.0	2.7	1.3	5.7	0.7	1.3	7
Aliskiren		5.0	12.3	5.0	2.1	12.1	1.0	2.1	5
Acetylsalicylic acid^*^	Analgesics	2.3	30.6	2.3	1.7	3.1	0.9	1.7	45
Naproxen		2.1	18.1	2.1	1.5	3.0	0.8	1.5	33
Rizatriptan^*^		6.5	31.7	6.5	3.2	13.0	1.3	3.2	8
Erythromycin^*^	Anti-acne preparations	4.8	23.7	4.9	2.5	9.3	1.2	2.5	9
Iron	Antianemic preparations	2.5	9.3	2.5	1.4	4.4	0.7	1.4	12
Ferric carboxymaltose^*^		6.4	13.2	6.4	2.4	17.1	1.0	2.4	4
Iron sucrose		4.9	8.8	4.9	1.8	13.1	0.9	1.8	4
Ferric oxyhydroxide^*^		11.1	18.4	11.1	3.6	34.5	1.1	3.6	3
Iron saccharate		4.9	5.8	4.9	1.6	15.1	0.7	1.6	3
Clarithromycin^*^	Antibacterials for systemic use	19.4	1309.2	19.4	15.5	24.3	3.4	14.9	79
Metronidazole^*^		13.6	820.1	13.6	10.8	17.1	3.0	10.4	74
Amoxicillin^*^		6.0	273.8	6.0	4.7	7.6	2.0	4.6	69
Linezolid^*^		22.1	1135.3	22.1	17.1	28.7	3.4	16.6	59
Ciprofloxacin^*^		3.6	61.4	3.6	2.6	5.1	1.3	2.6	34
Azithromycin^*^		5.5	108.8	5.5	3.9	7.9	1.7	3.8	31
Doxycycline^*^		4.7	70.8	4.7	3.2	6.9	1.5	3.2	26
Moxifloxacin^*^		5.7	79.0	5.7	3.7	8.6	1.6	3.7	22
Amikacin^*^		13.0	174.5	13.0	8.1	20.9	2.3	8.0	17
Clindamycin^*^		4.3	40.0	4.3	2.7	7.0	1.3	2.7	17
Clavulanate^*^		4.6	40.8	4.6	2.8	7.5	1.3	2.8	16
Cefuroxime^*^		11.7	126.2	11.8	7.0	19.9	2.1	6.9	14
Tetracycline^*^		20.0	232.0	20.0	11.8	33.9	2.5	11.7	14
Imipenem^*^		5.0	12.3	5.0	2.1	12.1	1.0	2.1	5
Omadacycline^*^		48.0	185.2	48.4	20.1	116.9	2.3	19.8	5
Cefdinir^*^		5.7	11.2	5.7	2.1	15.3	0.9	2.1	4
Penicillin v		5.0	9.1	5.0	1.9	13.3	0.9	1.9	4
Sulbactam		5.2	6.4	5.2	1.7	16.1	0.8	1.7	3
Telithromycin^*^		8.1	12.3	8.1	2.6	25.3	1.0	2.6	3
Bismuth subsalicylate^*^	Antidiarrheals, intestinal anti-inflammatory/anti-infective agents	31.4	408.9	31.6	19.0	52.5	3.0	18.7	15
Sulfasalazine		2.2	8.6	2.2	1.3	3.7	0.7	1.3	15
Loperamide		2.3	8.9	2.3	1.4	3.9	0.7	1.4	14
Nystatin^*^		8.9	90.4	9.0	5.3	15.2	1.9	5.3	14
Beclomethasone dipropionate^*^		5.3	37.4	5.3	3.0	9.3	1.4	3.0	12
Scopolamine butylbromide^*^	Antiemetics and antinauseants	31.6	265.5	31.8	17.0	59.3	2.7	16.9	10
Clonazepam	Antiepileptics	2.0	15.2	2.0	1.4	2.9	0.7	1.4	31
Fluconazole^*^	Antifungals for dermatological use	3.4	24.1	3.4	2.1	5.5	1.1	2.0	16
Amorolfine^*^		113.6	231.4	115.9	36.9	363.9	2.2	36.1	3
Eltrombopag olamine	Antihemorrhagics	3.9	8.0	3.9	1.6	9.3	0.8	1.6	5
Fexofenadine^*^	Antihistamines for systemic use	3.3	39.8	3.3	2.3	4.9	1.2	2.3	26
Cyclizine^*^		7.5	11.0	7.5	2.4	23.3	1.0	2.4	3
Clonidine^*^	Antihypertensives	3.3	24.6	3.3	2.0	5.3	1.1	2.0	17
Clofazimin^*^e	Antimycobacterials	7.0	15.1	7.1	2.6	18.8	1.1	2.6	4
Pyrazinamide		3.4	4.7	3.4	1.3	9.1	0.7	1.3	4
Cycloserine^*^		22.7	83.1	22.8	9.5	55.0	1.9	9.4	5
Capecitabine^*^	Antineoplastic agents	3.9	87.3	3.9	2.9	5.2	1.4	2.8	43
Niraparib^*^		6.5	82.5	6.5	4.1	10.2	1.7	4.1	19
Sunitinib^*^		3.9	22.8	3.9	2.2	6.9	1.1	2.2	12
Cabozantinib		2.0	4.1	2.0	1.1	3.7	0.5	1.1	10
Sorafenib		2.8	6.6	2.8	1.4	6.0	0.7	1.3	7
Sunitinib malate		2.6	4.5	2.6	1.2	5.9	0.5	1.2	6
Procarbazine^*^		6.3	17.4	6.3	2.6	15.3	1.1	2.6	5
Atovaquone	Antiprotozoals	4.6	7.9	4.6	1.7	12.2	0.8	1.7	4
Doxepin	Antipruritics	3.6	7.0	3.6	1.5	8.7	0.8	1.5	5
Calcipotriol^*^	Antipsoriatics	11.3	65.0	11.3	5.7	22.7	1.7	5.6	8
Triclosan^*^	Antiseptics and disinfectants	30.0	57.6	30.2	9.7	93.9	1.6	9.6	3
Ribavirin^*^	Antivirals for systemic use	4.2	96.7	4.2	3.1	5.7	1.5	3.0	42
Telaprevir^*^		4.8	44.0	4.8	2.9	7.8	1.4	2.9	16
Oseltamivir		2.8	6.5	2.8	1.3	5.9	0.7	1.3	7
Boceprevir^*^		6.0	16.1	6.0	2.5	14.4	1.1	2.5	5
Atenolol	Beta blocking agents	2.4	19.2	2.4	1.6	3.5	0.8	1.6	26
Cetylpyridinium^*^	Blood substitutes and perfusion solutions	172.6	857.3	178.1	78.9	401.9	3.3	76.3	6
Lidocaine	Cardiac therapy	3.0	22.4	3.0	1.9	4.6	1.0	1.9	19
Digoxin		2.8	17.6	2.8	1.7	4.5	0.9	1.7	17
Cortisone	Corticosteroids for systemic use	3.4	4.7	3.5	1.3	9.2	0.7	1.3	4
Mometasone	Corticosteroids, dermatological preparations	2.2	5.9	2.2	1.2	3.9	0.6	1.2	11
Desoximetasone^*^		11.1	81.5	11.1	6.0	20.7	1.9	5.9	10
Dextromethorphan	Cough and cold preparations	2.6	8.0	2.6	1.4	4.8	0.7	1.4	10
Guaifenesin		2.6	8.2	2.6	1.4	4.8	0.7	1.4	10
Glycopyrronium^*^	Dermatological preparations	10.5	144.2	10.5	6.6	16.7	2.1	6.6	18
Finasteride		2.7	11.2	2.7	1.5	4.8	0.7	1.5	12
Glycopyrrolate		3.3	4.2	3.3	1.2	8.7	0.6	1.2	4
Iodide I^131*^	Diagnostic radiopharmaceuticals	37.2	106.8	37.4	14.0	100.1	2.0	13.9	4
Sodium iodide i-131^*^		24.0	66.5	24.1	9.0	64.4	1.7	9.0	4
Citric acid^*^	Digestives	13.6	46.2	13.6	5.6	32.7	1.6	5.6	5
Hydrochlorothiazide	Diuretics	2.3	38.0	2.3	1.7	2.9	0.9	1.7	57
Indapamide^*^		6.0	44.5	6.0	3.4	10.5	1.5	3.4	12
Triamterene^*^		4.8	29.6	4.8	2.7	8.8	1.3	2.7	11
Omeprazole	Drugs for acid related disorders	2.1	34.7	2.1	1.6	2.7	0.8	1.6	66
Lansoprazole^*^		2.7	45.8	2.7	2.0	3.6	1.1	2.0	45
Esomeprazole		2.6	39.8	2.6	1.9	3.5	1.0	1.9	43
Famotidine		2.1	7.5	2.1	1.3	3.5	0.6	1.3	15
Bismuth sub citrate potassium^*^		85.8	752.4	87.1	46.6	162.9	3.4	45.7	10
Aluminum		5.5	7.0	5.5	1.8	17.1	0.8	1.8	3
Naloxone^*^	Drugs for constipation	3.9	40.5	3.9	2.5	6.1	1.3	2.5	20
Tiotropium^*^	Drugs for obstructive airway diseases	5.0	203.7	5.0	3.9	6.4	1.8	3.9	66
Formoterol^*^		4.8	183.8	4.8	3.8	6.2	1.7	3.7	64
Montelukast^*^		3.6	82.2	3.7	2.7	4.9	1.4	2.7	45
Vilanterol^*^		3.8	57.6	3.8	2.6	5.4	1.3	2.6	30
Omalizumab		2.7	21.7	2.7	1.8	4.1	0.9	1.8	22
Umeclidinium^*^		3.2	24.5	3.2	2.0	5.1	1.0	2.0	18
Theophylline^*^		13.8	176.5	13.9	8.5	22.7	2.3	8.4	16
Indacaterol^*^		4.7	11.3	4.8	2.0	11.4	0.9	2.0	5
Alendronate	Drugs for treatment of bone diseases	2.3	13.7	2.3	1.5	3.6	0.8	1.4	20
Risedronic acid		3.7	7.2	3.7	1.5	8.8	0.8	1.5	5
Peginterferon alfa-2a^*^	Immunostimulants	5.3	113.8	5.3	3.8	7.5	1.7	3.8	34
Peginterferon alfa-2b		2.8	5.2	2.8	1.3	6.2	0.7	1.3	6
Selenium^*^	Mineral supplements	21.1	39.0	21.2	6.8	65.8	1.4	6.8	3
Budesonide^*^	Nasal preparations	4.8	213.8	4.8	3.8	6.0	1.8	3.7	75
Fluticasone^*^		4.4	189.9	4.4	3.5	5.6	1.7	3.4	75
Salbutamol		2.2	45.3	2.2	1.8	2.8	0.9	1.7	72
Salmeterol^*^		5.5	246.3	5.5	4.3	7.0	1.9	4.2	70
Fluticasone propionate^*^		3.5	114.1	3.5	2.7	4.4	1.4	2.7	68
Fluticasone furoate^*^		3.9	60.9	3.9	2.7	5.6	1.4	2.7	30
Ipratropium bromide		2.7	12.4	2.7	1.6	4.7	0.8	1.6	13
Salmeterol xinafoate		2.1	6.0	2.1	1.2	3.7	0.6	1.2	12
Ipratropium		2.8	10.6	2.8	1.5	5.0	0.8	1.5	11
Phenylpropanolamine^*^		61.0	237.5	61.6	25.5	148.9	2.5	25.2	5
Nicotine^*^	Nervous system drugs	7.8	376.3	7.8	6.1	9.9	2.3	5.9	67
Paroxetine	Psychoanaleptics	2.4	19.5	2.4	1.6	3.5	0.8	1.6	26
Trazodone		2.1	11.7	2.2	1.4	3.3	0.7	1.4	21
Atomoxetine		2.7	7.8	2.7	1.4	5.2	0.7	1.4	9
Diazepam	Psycholeptics	2.2	13.1	2.2	1.4	3.3	0.7	1.4	23
Prochlorperazine		2.9	10.5	2.9	1.6	5.4	0.8	1.6	10
Haloperidol		2.1	4.0	2.1	1.1	4.0	0.5	1.1	9
Asenapine^*^		5.4	20.9	5.4	2.6	11.4	1.2	2.6	7
Temazepam		2.5	5.0	2.5	1.2	5.3	0.6	1.2	7
Triazolam		4.6	5.2	4.6	1.5	14.3	0.7	1.5	3
Estriol^*^	Sex hormones and modulators of the genital system	40.0	115.3	40.3	15.0	107.7	2.0	14.9	4
Chlorhexidine gluconate^*^	Stomatological preparations	84.8	3413.3	86.1	63.5	116.7	4.7	61.4	43
Sodium fluoride^*^		46.8	710.8	47.1	29.2	76.1	3.4	28.7	17
Minocycline		4.4	15.3	4.4	2.1	9.3	0.7	1.3	7
Chlorhexidine^*^		8.4	32.0	8.4	3.8	18.8	1.4	3.8	6
Miconazole^*^		5.5	17.9	5.5	2.5	12.3	1.1	2.5	6
Clotrimazole		4.0	6.3	4.0	1.5	10.8	0.8	1.5	4
Levothyroxine	Thyroid therapy	2.2	57.1	2.2	1.8	2.7	0.9	1.8	92
Thyroid porcine		4.8	8.6	4.8	1.8	12.9	0.9	1.8	4
Oxybutynin^*^	Urologicals	4.7	45.8	4.7	2.9	7.6	1.4	2.9	17
Tolterodine^*^		4.9	24.4	5.0	2.6	9.5	1.2	2.6	9
Apomorphine		4.4	9.9	4.4	1.8	10.5	0.9	1.8	5
Alfuzosin		3.5	4.8	3.5	1.3	9.3	0.7	1.3	4
Tiopronin^*^		9.2	14.5	9.2	3.0	28.7	1.0	3.0	3
Trospium^*^		6.4	8.8	6.4	2.1	19.9	0.9	2.1	3
Diltiazem	Vasoprotectives	3.0	26.4	3.0	1.9	4.5	1.0	1.9	22
Vitamin C	Vitamins	2.2	10.9	2.2	1.4	3.5	0.7	1.4	18
Vitamin B12		2.5	9.4	2.5	1.4	4.4	0.8	1.4	12
Vitamin E		2.8	4.1	2.8	1.2	6.7	0.6	1.2	5

*ATC* anatomical therapeutic chemical, *PRR* proportional reporting ratio, *χ*^2^ chi square, *ROR* reporting odds ratio, *IC* information component, *EBGM* empirical Bayes geometric mean, * Met all the criteria for signal detection.

### Signal detection measures for drug-associated strawberry tongue

Strawberry tongue showed a markedly different drug association profile compared to general tongue discoloration (Table 3). Seven drugs demonstrated significant signals, with analgesics being particularly prominent: Non-steroidal anti-inflammatory drugs (NSAIDs) and analgesics: acetaminophen, ibuprofen, aspirin; Antibiotics: amoxicillin, flucloxacillin; and immunomodulators: human immunoglobulin G, lamotrigine.

**Table 3:** Signal detection measures for drug-associated strawberry tongue.

Drugs	ATC system	PRR	*χ*^2^	ROR	Lower limit 95% CI of ROR	Upper limit 95% CI of ROR	IC025	EBGM05	Number of cases
Acetaminophen*	Analgesics	8.7	30.2	8.7	3.7	20.5	1.2	2.9	7
Ibuprofen*		17.5	50.8	17.5	6.6	45.9	1.5	5.5	5
Aspirin*		8.8	17.3	8.8	3.0	25.3	1.0	2.7	4
Amoxicillin*	Antibacterials for systemic use	22.8	38.0	22.8	6.9	75.6	1.3	6.2	3
Flucloxacillin*		706.6	1848.6	707.5	245.3	2040.3	3.2	210.0	4
Human immunoglobulin G*	Immune sera and immunoglobulins	64.9	164.2	64.9	22.5	186.9	2.0	19.4	4
Lamotrigine*	Antiepileptics	26.4	63.3	26.4	9.2	76.1	1.6	7.9	4

*ATC* anatomical therapeutic chemical, *PRR* proportional reporting ratio, *χ*^2^ chi square, *ROR* reporting odds ratio, *IC* information component, *EBGM* empirical Bayes geometric mean, * Met all the criteria for signal detection.

### Signal detection measures for drug-associated black hairy tongue

Black hairy tongue demonstrated associations with a diverse range of therapeutic classes (Table 4), including: Gastrointestinal drugs: esomeprazole (proton pump inhibitor); Cardiovascular medications: simvastatin, atenolol; Psychiatric medications: paroxetine, quetiapine; and Respiratory drugs: fluticasone.

**Table 4:** Signal detection measures for drug-associated black hairy tongue.

Drugs	ATC system	PRR	*χ*^2^	ROR	Lower limit 95% CI of ROR	Upper limit 95% CI of ROR	IC025	EBGM05	Number of cases
Amoxicillin^*^	Antibacterials for systemic use	8.9	13.3	8.9	2.8	28.4	1.0	2.7	3
Meclizine^*^	Antihistamines for systemic use	78.2	150.8	78.2	24.6	248.9	2.0	23.4	3
Atenolol^*^	Beta blocking agents	9.6	14.5	9.6	3.0	30.4	1.0	2.9	3
Esomeprazole^*^	Drugs for acid related disorders	10.8	32.4	10.8	4.3	26.8	1.3	4.0	5
Simvastatin^*^	Lipid modifying agents	6.0	11.3	6.0	2.2	16.5	0.9	2.1	4
Fluticasone propionate^*^	Nasal preparations	11.0	41.0	11.0	4.8	25.5	1.4	4.4	6
Nicotine^*^	Nervous system drugs	20.5	68.5	20.5	8.2	50.9	1.7	7.6	5
Paroxetine^*^	Psychoanaleptics	9.6	14.7	9.6	3.0	30.7	1.0	2.9	3
Quetiapine	Psycholeptics	5.0	5.7	5.0	1.6	15.8	0.7	1.5	3
Tamsulosin^*^	Urologicals	10.4	16.1	10.4	3.3	33.0	1.0	3.1	3

*ATC* anatomical therapeutic chemical, *PRR* proportional reporting ratio, *χ*^2^ chi square, *ROR* reporting odds ratio, *IC* information component, *EBGM* empirical Bayes geometric mean, * Met all the criteria for signal detection.

## Discussion

### Key findings

The analysis of 2,352 unique reports from the FAERS revealed significant associations between various drugs and tongue-related adverse events, including tongue discoloration, strawberry tongue, and black hairy tongue. Middle-aged individuals were more commonly affected by tooth discoloration and black hairy tongue, while strawberry tongue was predominantly observed in adolescents and children. A female preponderance was noted across all adverse events. Several drugs, such as clarithromycin, metronidazole, linezolid, and amoxicillin, were linked to positive signals for tongue discoloration in both frequentist and Bayesian analyses, whereas analgesics, flucloxacillin, amoxicillin, lamotrigine, and human immunoglobulin G were consistently associated with strawberry tongue. Strikingly, meclizine, atenolol, amoxicillin, esomeprazole, simvastatin, fluticasone, paroxetine, and tamsulosin showed positive signals for black hairy tongue. These findings highlight the potential drug-related risks for specific tongue conditions and underscore the need for clinical awareness and monitoring.

### Comparison with existing literature

Tongue discoloration can be classified as either intrinsic or extrinsic based on its underlying cause. Intrinsic discoloration arises from systemic factors that affect the internal tissues of the tongue. This type of discoloration may result from conditions such as jaundice, where an excess of bilirubin leads to a yellowish hue, or vitamin deficiencies that can cause changes in tongue color and texture. On the other hand, extrinsic discoloration is typically due to external factors that stain the surface of the tongue. Common causes include the use of tobacco, certain foods and beverages (like coffee or red wine), and medications that cause a buildup of pigments or debris on the tongue.

Several antimicrobial drugs, drugs used for treating gastrointestinal tract and nervous system disorders, iron preparations, dermatological preparations, vitamins, and topical preparations used in mouth were observed with tongue discoloration in the present study. We observed that several vitamins, radio pharmaceuticals, and topical mouth preparations have been identified with new signals for tongue discoloration. Patients developing tongue discoloration associated with drug therapy should be advised to undertake tongue scraping thrice a day, maintain proper hydration, and comply with appropriate oral hygienic practices [15]. Hence, it is a paradox that mouth washes were used for treating tongue discoloration, as well as they are implicated as causative drugs. Further mouth washes contain several excipients other than active ingredients, such as alcohol (flavor enhancer and solubilizer), preservatives (benzalkonium chloride, parabens, benzoates, and sorbates), surfactants, humectants (for improving the viscosity), and coloring agents [16]. Hence, mouthwashes without coloring agents should be preferred for those with history of tongue or tooth discoloration. In addition, they should consult their healthcare provider to evaluate the possibility of discontinuing the causative drug. If necessary, alternative medications that do not contribute to tongue discoloration should be considered, and close monitoring of the patient’s response to the new treatment is essential [17]. The decision to discontinue the causative medication requires careful risk-benefit assessment. For essential medications (such as cardiovascular drugs or long-term antibiotics), where immediate discontinuation might pose greater risks, watchful waiting with enhanced oral hygiene measures may be appropriate. However, for non-essential medications or where alternative therapies are readily available, drug discontinuation could be considered. The timing of discontinuation should also account for the severity of discoloration, patient distress, and the availability of suitable alternatives. The decision on therapeutic rechallenge should be considered based on the severity of tongue discoloration, availability of alternatives, and patient preference.

Tongue discoloration, particularly black hairy tongue, has been well-documented in association with antimicrobial drugs, particularly antibiotics like tetracyclines, metronidazole, and linezolid. Prior studies have linked these drugs to black hairy tongue due to their potential to alter the oral microbiota, leading to overgrowth of pigmented bacteria or fungi on the tongue surface [18]. Our study corroborates these associations, identifying a wide range of antimicrobials with positive signals, including erythromycin, clarithromycin, and azithromycin, which are consistent with findings from earlier reports. Furthermore, antimicrobials such as cefdinir, known for causing red tongue discoloration, were also flagged by both frequentist and Bayesian methods, reinforcing prior evidence. The association of black hairy tongue with nervous system drugs, such as antipsychotics and antidepressants, is noteworthy. This supports prior literature where psychotropic medications, such as paroxetine and quetiapine, have been implicated in oral side effects, including tongue discoloration, due to their anticholinergic properties, leading to dry mouth and altered oral flora [19, 20]. Additionally, the detection of nicotine as a contributing factor to both black hairy tongue and tongue discoloration aligns with established knowledge of nicotine’s role in modifying oral hygiene and predisposing individuals to oral conditions like black hairy tongue. Additionally, their anticholinergic activity (also observed with meclizine, antihistamine) leads to reduced salivary production, conducive to bacterial overgrowth and excessive keratin accumulation in the tongue.

Interestingly, we also detected a signal for strawberry tongue with drugs like acetaminophen, ibuprofen, and amoxicillin. Strawberry tongue is frequently observed in conditions like Kawasaki disease or scarlet fever, but drug-induced strawberry tongue remains under-reported. Our results suggest that common over-the-counter medications like acetaminophen and ibuprofen may contribute to this phenomenon, adding to the body of evidence that non-infectious causes should be considered in clinical assessments. Local vasodilation associated with altered inflammatory response is the likely explanation underlying strawberry tongue.

Risk factors identified previously, like older age, smoking, and various diseases [4], were also represented in our cohort. The higher prevalence of tooth discoloration and black hairy tongue in middle age may relate to accumulating comorbidities and polypharmacy over time. Interestingly, we found strawberry tongue more common in younger groups, consistent with reports linking it to infections in children [21]. We also observed female preponderance across all types of drug-associated tongue discoloration merits careful consideration. Several factors may contribute to this gender disparity. First, women are generally more likely to seek healthcare services and report adverse drug events compared to men, potentially leading to reporting bias in spontaneous reporting systems like FAERS [22]. Second, women typically have higher healthcare utilization rates and consequently greater exposure to medications [23]. Third, sex-based differences in pharmacokinetics and pharmacodynamics might play a role, women generally have lower body weight, different fat distribution, and variations in drug-metabolizing enzymes compared to men, potentially affecting drug concentrations and adverse effect risks [24]. Additionally, hormonal influences on immune responses and drug metabolism might contribute to increased susceptibility to drug reactions in females. However, these explanations remain hypothetical within the context of tongue discoloration, as our study design cannot establish causal relationships. Future research should specifically investigate these gender-related differences through controlled studies. Overall, our findings complement prior literature while offering new signals for continued evaluation. Further research is warranted to explore the mechanistic pathways leading to these adverse events and to develop preventive strategies.

### Strengths and limitations

This study benefits from a large sample size drawn from the FAERS database, allowing detection of potential signals across many drug classes. The use of multiple statistical methods enhances confidence in signals emerging from both frequentist and Bayesian approaches. However, several limitations should be considered. FAERS data rely on spontaneous reporting, which can be affected by reporting bias. Under-reporting of less severe events is common. Causality cannot be proven due to the non-interventional nature of pharmacovigilance databases. Confounding by indication may influence some associations, as certain disorders preferentially receive treatments. Details on dose, duration, and treatment compliance were limited. Our deduplication strategy, which retained only the most recent report, represents a potential limitation. While this approach helps avoid duplicate counting, it may have inadvertently excluded valuable follow-up information contained in earlier reports. This limitation could particularly impact our understanding of the outcomes and clinical course of tongue discoloration. Future studies should consider more comprehensive deduplication strategies that preserve longitudinal information from follow-up reports while maintaining data integrity. Additionally, validation studies comparing different deduplication methodologies in pharmacovigilance signal detection would be valuable for establishing best practices in FAERS analyses. The methodological choice of excluding cases with secondary suspected drugs was made in alignment with our primary objective of signal detection. Pharmacovigilance studies using AERS databases are designed to identify signals rather than establish causality. Our focus on primary suspect drugs aimed to optimize signal detection sensitivity. The analysis of drug interactions in polypharmacy requires different pharmacovigilance approaches, distinct statistical methodologies, and substantially larger sample sizes than traditional signal detection. Such interaction analyses would constitute a separate research question that can be explored in future studies. Lastly, the study was unable to evaluate severity or recurrence of events. Further research with prospective data collection can help address some of these limitations and validate priority signals identified here.

## Conclusion

In conclusion, this pharmacovigilance disproportionality analysis of the FAERS database revealed several significant drug-tongue discoloration associations meriting further investigation. Key signals involved commonly prescribed antimicrobials, gastrointestinal drugs, analgesics, stomatological products, and dermatologic medications. Differential patterns emerged depending on the specific tongue condition. While establishing causality remains challenging due to limitations of spontaneous reporting data, consistency across statistical methods enhances confidence in priority signals. Our findings show considerable implications for guiding clinical practice by highlighting potential adverse reactions requiring closer monitoring. They also inform the selection of safer treatment alternatives, when possible, substitutes exist. Further research through prospective observational studies can help validate signals identified here and elucidate attributable risks. Overall, this work underscores the importance for continued drug safety surveillance to optimize benefit-risk assessment and reduce preventable tongue disorders of diverse etiologies.

## Data Availability

The data is available in the USFDA AERS web-portal that can be accessed as follows: https://fis.fda.gov/sense/app/95239e26-e0be-42d9-a960-9a5f7f1c25ee/sheet/7a47a261-d58b-4203-a8aa-6d3021737452/state/analysis
